# Clinical Manifestations, Treatment, and Outcome of Hospitalized Patients with *Plasmodium vivax* Malaria in Two Indian States: A Retrospective Study

**DOI:** 10.1155/2013/341862

**Published:** 2013-12-29

**Authors:** Jagjit Singh, Bhargav Purohit, Anupama Desai, Lalita Savardekar, Preeti Shanbag, Nilima Kshirsagar

**Affiliations:** ^1^Department of Pharmacology, Government Medical College and Hospital, Chandigarh 160035, India; ^2^Department of Pharmacology, GMERS Medical College, Sola, Ahmedabad, Gujarat 382012, India; ^3^Department of Pharmacology, Surat Municipal Institute of Medical Sciences & Research (SMIMER), Surat, Gujarat 395010, India; ^4^National Institute of Research in Reproductive Health, Parel, Mumbai 400012, India; ^5^ESI-PGIMSR & MGM Hospital, Parel, Mumbai 400012, India; ^6^National Chair Clinical Pharmacology, Indian Council of Medical Research, ESI-PGIMSR & MGM Hospital, Parel, Mumbai 400012, India

## Abstract

This was a retrospective study done on 110 patients hospitalized with *P. vivax* malaria in three medical college hospitals, one in the union territory of Chandigarh and the other two in Gujarat, that is, Ahmedabad and Surat. The clinical presentation, treatment, and outcome were recorded. As per WHO criteria for severity, 19 of 110 patients had severe disease—six patients had clinical jaundice with hepatic dysfunction, three patients had severe anemia, three had spontaneous bleeding, two had acute respiratory distress syndrome, and one had cerebral malaria, hyperparasitemia, renal failure, circulatory collapse, and metabolic acidosis. All patients with severe *P. vivax* malaria survived, but one child with cerebral malaria had neurological sequelae. There was wide variation in the antimalarial treatment received at the three centres. *Plasmodium vivax* malaria can no longer be considered a benign condition. WHO guidelines for treatment of *P. vivax* malaria need to be reinforced.

## 1. Introduction

India accounts for 77% of the total malaria in south-east Asia with *Plasmodium vivax* being responsible for more than 50% of the cases. Although earlier regarded as causing a benign infection, there is increasing evidence that the overall burden, economic impact, and severity of *P. vivax* have been underestimated [1]. There are only three large studies from India which describe the whole spectrum of severe clinical manifestations of *P. vivax* malaria [2–4].

Also, malaria case management remains a vital component of malaria control strategies. The WHO guidelines for the treatment of malaria recommend chloroquine (except in areas of chloroquine resistance) as first-line treatment for uncomplicated *P. vivax* malaria with 14 days of primaquine for radical cure. The WHO guidelines for the treatment of malaria recommend that intravenous artesunate should be used for the treatment of severe malaria (*falciparum* and *vivax*) for a minimum period of 24 hours. Following initial parenteral treatment, once the patient can tolerate oral therapy, it is essential to continue and complete treatment with an effective oral antimalarial using a full course of an effective ACT (artesunate plus amodiaquine or artemether plus lumefantrine or dihydroartemisinin plus piperaquine) or artesunate (plus clindamycin or doxycycline) or quinine (plus clindamycin or doxycycline) [5].

The present study was done to evaluate the clinical manifestations and outcome in patients hospitalized with *P. vivax* malaria and whether WHO guidelines for treatment of *P. vivax* malaria were being adhered to.

## 2. Methods 

### 2.1. Design and Setting of Study

This was a retrospective study done using data collected from the medical records of three tertiary care centres. One of the hospitals was in Chandigarh, a union territory in the northern region of India, whereas two of the hospitals were in Gujarat (Sola, Ahmedabad and Surat), a state in the western region of India. Records of patients admitted to the ward or intensive care unit of the respective hospitals, from May to December 2012, with a diagnosis of *P. vivax* malaria, were studied. The period from May to December was chosen since it coincides with the monsoons in India, a period when transmission of malaria is at its highest [6, 7].

### 2.2. Target Population

Adults and children, with smear-positive *P. vivax* malaria were included in the study. Patients of *P. falciparum* malaria, those with mixed infections and those with other coexisting infections like typhoid fever, dengue, and leptospirosis, were excluded.

### 2.3. Tool for Data Collection

A case record form was developed and used to retrieve data from the inpatient records. The first three authors were responsible for extracting data from the medical records of their respective hospitals.

### 2.4. Ethical Issues

Ethics committee approval was obtained from all three hospitals for retrieval of data from the medical records. The confidentiality of the patients' record and identity of the treating physician was adequately protected using codes.

### 2.5. Data Extraction

Information extracted from the records included patient's age, sex, the presenting complaints, findings on clinical examination, hematological and biochemical investigations, treatment given, course in the hospital, and outcome.

In the absence of guidelines to classify severe *P. vivax* malaria, the WHO guidelines for classification of severe *P. falciparum* malaria were used to identify patients with severe disease [6].

### 2.6. Data Management

The data was pooled at the coordinating centre at the National Institute for Research in Reproductive Health, Mumbai. Data entry and analysis were done using Microsoft Excel 2007. Descriptive statistics in terms of proportions for qualitative variables were applied for analysis of data.

## 3. Results

### 3.1. Patient Characteristics

Medical records of a total of 110 patients hospitalized for *P. vivax* malaria in the three centres were studied. The ages of the patients are summarized in Table 1. Twenty-four of these 110 patients were children less than 12 years of age.

### 3.2. Clinical Features

The presenting symptoms are summarised in Table 2. Fever was the commonest symptom being present in all the patients followed by chills which were present in 86 of 110 patients.

### 3.3. Severe Disease

Nineteen of 110 patients (17.2%) hospitalized with *P. vivax* malaria had severe disease (Tables 3 and 4). Jaundice with evidence of other vital organ dysfunction was seen in eight patients. Severe anemia was seen in five patients. Though 38 patients had thrombocytopenia, only three had bleeding manifestations (epistaxis-1, hematemesis, and malena-2).

### 3.4. Treatment

The treatment and outcome of patients with severe disease is given in Table 5.

The five patients with severe anemia received packed cell transfusions. Three patients with thrombocytopenia (platelet counts of 12,000/cu·mm, 26,000/cu·mm, and 23,000/cu·mm) had bleeding manifestations and received platelet transfusions. Two patients with ARDS required ventilation. The one patient with acute renal failure required dialysis, with indications being anuria and hyperkalemia.

### 3.5. Outcome

All patients with *P. vivax* malaria including those with severe disease survived. One child with cerebral malaria had sequelae in the form of blindness which was still present at one-month followup.

## 4. Discussion 

Nineteen of 110 (17.2%) hospitalized patients with *P. vivax* malaria had evidence of severe disease. Only three large studies describing the whole spectrum of severe manifestations with *P. vivax* malaria could be identified from India; two were from Bikaner by Kochar et al. [2, 3], while the third was from Mumbai by Nadkar et al. [4]. Severe disease was seen in 63.1% of children as compared to 8.8% of adults hospitalized for *P. vivax* malaria in Bikaner. The criteria for hospitalizing patients with *P. vivax* malaria were not well defined in our study or in the published studies and were at the discretion of the admitting physician.

Severe anemia (hemoglobin < 5 G/dL) necessitating packed red cell transfusion was seen in five of nineteen patients with severe malaria in this study. The low parasite biomass of *P. vivax* indicates that severe anemia is not due to destruction of infected RBCs alone. Malariotherapy studies have shown that, for every infected RBC destroyed during *vivax* infection, 32 noninfected RBCs are removed from the circulation, compared to the loss of 8 RBCs for every infected erythrocyte in *falciparum* malaria. Cytokine-related dyserythropoiesis also probably contributes to anemia [8].

Thrombocytopenia was seen in 34.55% of patients hospitalized with *vivax* malaria. Bleeding manifestations necessitating platelet transfusions were seen in only three patients. The frequency of thrombocytopenia, that is, platelet count <1,50,000/cu·mm, in malaria ranges from 24% to 94% in the literature and this frequency is not different in the two species. However, occurrence of bleeding even in severe illness is low and a conservative approach is adopted in most cases. Platelet counts usually revert to normal after effective antimalarial treatment [9].

Cerebral malaria was seen in three of nineteen patients with severe *vivax* malaria in our study. Cytoadherence phenomena are believed to be central to the etiology of cerebral malaria in *falciparum* infection, but their role in *P. vivax *malaria remains unclear [10].

One patient each from Chandigarh and Surat had acute respiratory distress syndrome (ARDS) and required ventilatory support. ARDS is considered to be the most severe form of acute lung injury in malaria and has been reported most commonly with *P. falciparum* malaria. Case reports of ARDS with *P. vivax* malaria are also being increasingly reported [11].

Clinical jaundice with hepatic dysfunction was seen in eight of nineteen patients with severe *vivax* malaria. Hepatic dysfunction has been also described in 20–58% of patients with *vivax* malaria in Bikaner and Mumbai [2–4].

The WHO guidelines recommend that severe *P. vivax* malaria be treated along the lines of severe *falciparum* malaria with artemisinin-based combination therapies (ACT) [5].

There was wide variation in the therapy for severe disease in the three centres. Chloroquine was used as the antimalarial of choice in Sola even in patients with severe disease. In Surat, both artesunate and chloroquine were used in patients with severe *P. vivax* malaria, whereas in Chandigarh artesunate only was used in four patients. ACT was used in four patients (three in Chandigarh and one in Surat). The rationale for using antimalarial combination therapy is the simultaneous use of two or more blood schizontocidal medicines with independent modes of action and, thus, different biochemical targets in the parasite. This is postulated to prevent or delay the appearance of resistance [5].

Drug resistance to the first-line drug chloroquine has been reported from Papua New Guinea, Indonesia, parts of Asia including India, and South America [12–14]. Chloroquine should be used as the drug of first choice in vivax malaria due to the low prevalence of 1-2% resistance in these patients. This is particularly important for countries like India where between 60 and 70% of the cases are caused by *vivax* [1]. However, surveillance for drug resistance is recommended and drug policy should be based on surveillance data.

WHO guidelines recommend primaquine for 14 days for radical cure in *P. vivax* malaria [5]. None of the patients in Chandigarh received primaquine due to temporary nonavailability of kits for estimation of G6PD level. All hospitalized patients with *P. vivax* malaria in Sola and Surat received primaquine in the correct dosage.

There were no deaths in any of the patients hospitalized with *P. vivax* malaria at any of the three centres. One child with cerebral malaria in Chandigarh had neurological sequelae at discharge. A mortality of 6% was noted in children in Bikaner, whereas it was 5% and 0.9% in adults in Bikaner and Mumbai, respectively [2–4].

The limitations of this study are as follows: it is a retrospective study with a small sample size and uses data retrieved from the medical records. The problem of mixed infection has not been addressed adequately since this was a retrospective study and PCR is not done routinely at the three centres. A prospective multicentric study with an additional centre in Mumbai is under way and will no doubt clarify the picture better.

## 5. Conclusions

*Plasmodium vivax* can result in severe disease and can no longer be considered to be a benign condition in India. There is considerable variation in the treatment of *P. vivax* malaria. Adherence to WHO guidelines for treatment is recommended.

## Figures and Tables

**Table 1:** Age of patients.

Age in years	Number of patients
0–12	24
13–20	21
21–40	43
41–60	17
>60	5

Total	110

**Table 2:** Symptoms of patients hospitalized with *P*. *vivax* malaria in the three centres.

Symptom	Number (*n* = 110)
Fever	110
Chills	86
Headache	53
Bodyache	44
Seizures	03

**Table 3:** Hematological parameters of patients with severe *vivax * malaria.

No.	Hb (g/dL)	Total count 10^3^/mm^3^	Platelet count 10^3^/mm^3^	Blood sugar mg/dL	Blood urea nitrogen mg/dL	Serum creatinine mg/dL	Serum bilirubin mg/dL conjugated/unconjugated	Serum ALT IU/L
1	8.0	5.9	90	94	24	0.9	1.0/0.4	40
2	7.0	4.8	12	90	38	1.2	0.9/0.3	30
3	9.5	7.6	50	105	94	1.6	10.3/6.5	360
4	4.5	9.9	121	65	90	1.5	3.1/1.8	107
5	9.6	6.2	110	120	120	3.2	3.6/2.2	97
6	6.8	4.8	26	60	60	0.8	1.0/0.6	40
7	6.9	8.4	87	138	28	0.7	1.4/0.8	38
8	8.4	5.0	96	90	52	1.3	16.4/10.9	520
9	7.2	3.7	80	130	30	0.8	8.4/6.4	368
10	9.7	4.5	96	105	42	1.4	4.2/3.1	287
11	4.8	7.2	120	110	22	0.9	1.6/1.0	89
12	3.8	9.1	110	95	19	1.0	1.4/0.9	75
13	8.6	9.8	23	70	42	1.1	6.8/4.3	356
14	8.4	10.0	75	95	38	1.5	1.8/1.2	98
15	7.8	6.8	68	128	36	1.4	5.6/3.9	276
16	9.5	5.6	15	112	28	1.1	1.2/0.6	40
17	10.0	8.7	35	96	25	1.3	1.6/1.1	52
18	5.0	9.6	45	102	35	1.4	1.7/1.2	60
19	4.6	10.2	20	88	40	1.2	1.0/0.3	42

**Table 4:** Severe *P*. *vivax* malaria.

	Chandigarh	Sola, Ahmedabad	Surat	Total
Total number hospitalized with *P*. *vivax* malaria	22	59	29	110
Number with severe *P*. *vivax* malaria	7	6	6	19
Cerebral malaria	1	2	0	3
Circulatory collapse	1	0	0	1
Acute renal failure	1	0	0	1
Jaundice	2	2	4	8
Acute respiratory distress syndrome	1	0	1	2
Severe anemia	1	2	2	5
Thrombocytopenia	7	23	8	38
Hyperparasitemia	0	0	1	1
Metabolic acidosis	0	0	1	1

**Table 5:** Treatment and outcome of patients with severe disease.

	Chandigarh	Sola	Surat
Patients with severe malaria	7 (33.33%)	6 (10.17%)	6 (20.08%)
Artesunate alone	4	0	1
Chloroquine	0	5	5
Artemisinin-based combination therapy (ACT)	3	1	0
Primaquine	0	6	5
Packed cell transfusion	1	2	2
Platelet transfusion	3	0	0
Ventilation	2	0	1
Dialysis	1	0	0
Sequelae	1	0	0
Death	0	0	0
